# Panophthalmitis complicated with asymptomatic pyogenic liver abscess caused by *Klebsiella pneumoniae*: A case report

**DOI:** 10.1097/MD.0000000000042825

**Published:** 2025-06-06

**Authors:** Shu-Han Chuang, Cheng-Hsien Chang

**Affiliations:** a Division of General Practice, Department of Medical Education, Changhua Christian Hospital, Changhua, Taiwan; b Department of Ophthalmology, Changhua Christian Hospital, Changhua, Taiwan; c Department of Post-Baccalaureate Medicine, College of Medicine, National Chung Hsing University, Taichung, Taiwan.

**Keywords:** asymptomatic pyogenic liver abscess, *Klebsiella pneumoniae*, panophthalmitis

## Abstract

**Rationale:**

Panophthalmitis is a severe, vision-threatening condition that may result from endogenous spread of infection, often presenting with systemic symptoms. *Klebsiella pneumoniae* (Kp) is a known cause of endogenous panophthalmitis, typically associated with underlying conditions such as diabetes and often linked to pyogenic liver abscesses. However, cases presenting solely with ocular symptoms are exceedingly rare. This report presents a unique case of Kp panophthalmitis with a concurrent asymptomatic liver abscess, emphasizing the importance of systemic evaluation in atypical ocular infections.

**Patient concerns:**

A 66-year-old woman presented with a 3-day history of progressive vision loss, periorbital pain, swelling, and redness in the left eye. She denied fever, gastrointestinal symptoms, or other systemic complaints.

**Diagnoses:**

Imaging revealed panophthalmitis with retrobulbar infiltration. Blood tests showed leukocytosis and mildly elevated liver enzymes. A liver abscess was identified on ultrasound and computed tomography. Cultures from the eyelid abscess and liver aspiration both yielded *K. pneumoniae*.

**Interventions:**

The patient received empirical systemic antibiotics, intravitreal injections, and daily wound care. After 2 weeks without visual improvement, evisceration was performed. The liver abscess was aspirated and treated with antibiotics.

**Outcomes:**

Despite aggressive treatment, the left eye was nonviable and required evisceration. However, systemic infection was controlled, and the patient remained free of complications from the liver abscess. Notably, she exhibited no systemic symptoms throughout the disease course.

**Lessons:**

This case highlights the need for systemic evaluation in patients with severe ocular infections, even in the absence of systemic symptoms. It also underscores the potential for serious infections such as Kp-related liver abscess to present solely with ocular manifestations. Early suspicion and comprehensive assessment can prevent misdiagnosis and improve outcomes.

## 1. Introduction

Panophthalmitis is a severe ocular condition characterized by inflammation involving all structures of the globe with extension into the orbital structures, which often leads to vision loss.^[1,2]^ It may be associated with systemic infections, particularly in immunocompromised patients or those with underlying medical conditions, such as diabetes mellitus.^[2–5]^ Delayed or inadequate treatment can lead to serious consequences such as severe or permanent visual loss, enucleation, sepsis, or death. The etiology of panophthalmitis can be categorized into endogenous and exogenous.^[2,3]^ Exogenous causes most commonly include ocular trauma or iatrogenic infection. Endogenous spread occurs hematogenously, possibly from distant abscesses, gradually breaching the blood-ocular barrier.

Among them, endogenous *Klebsiella pneumoniae* (Kp) panophthalmitis is an emerging disease in Asia.^[3–6]^ It typically affects immunocompromised individuals with a generally poor visual prognosis. Timely diagnosis, early identification of the source of infection, followed by targeted therapy may offer a chance to salvage vision and control the infection. Here, we present a unique case of Kp panophthalmitis, which was complicated with an asymptomatic pyogenic liver abscess.

## 2. Case report

A 66-year-old female presented with vision loss in her left eye for 3 days, without any history of trauma or prior eye surgeries. Her only medical history was of heart valvular disease, which had been stable for about 20 years. The patient blamed the eye trouble on a mosquito bumping into her left eye while riding a bicycle 2 days before the onset of symptoms. Initially blurred vision in the left eye rapidly progressed from floaters to complete vision loss. Upon arrival at our hospital, she reported a headache, left periorbital pain, redness, and swelling. Otherwise, there were no fever, respiratory, or gastrointestinal symptoms.

**Figure 1.**
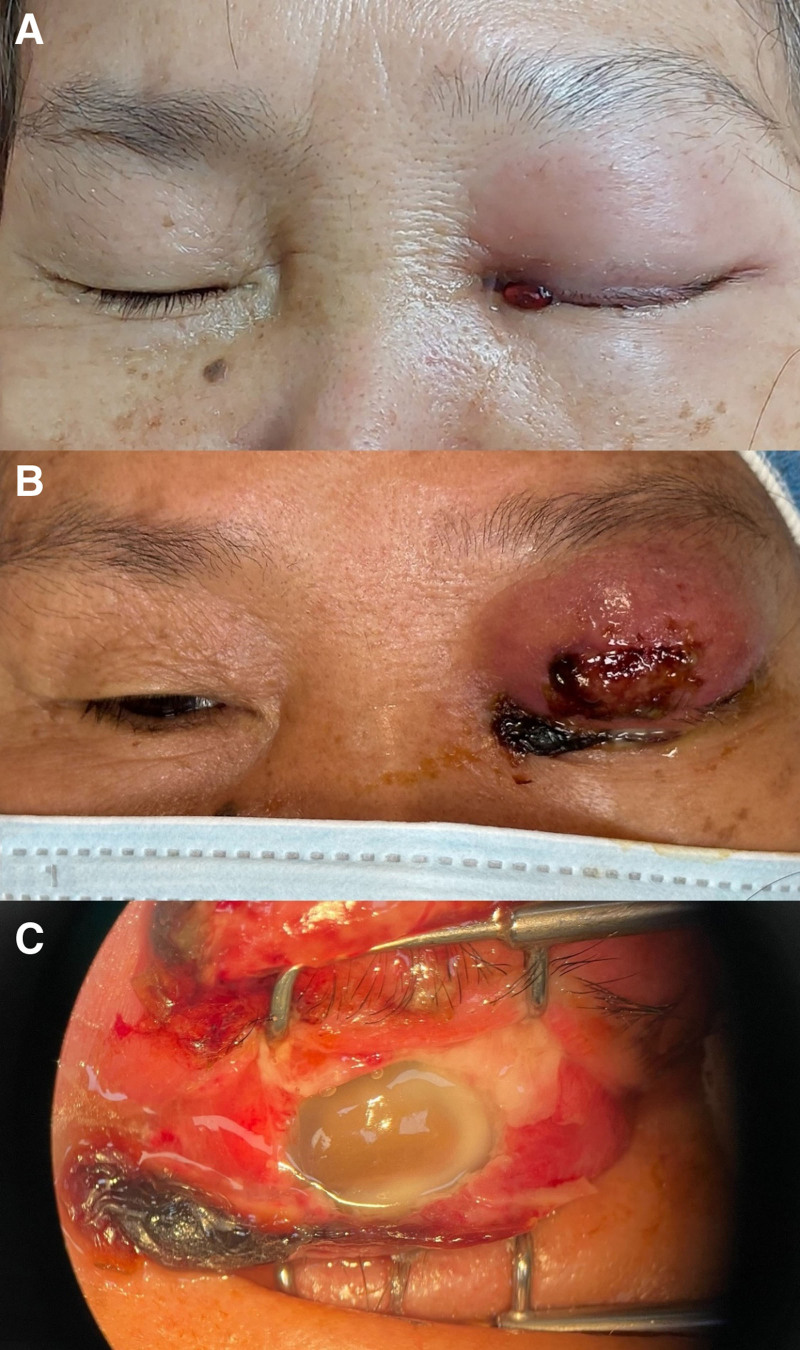
Appearance of the lesion upon admission (A) and after 1 week (B and C).

On examination, her left eye was a firm and tense globe with no light perception. The left eyelid was markedly swollen, red, and tender, with pus discharge. The cornea was diffusely edematous, and the conjunctiva was injected and chemotic (Fig. 1A). In the anterior chamber, there was a visible hypopyon and an exudative membrane.

**Figure 2.**
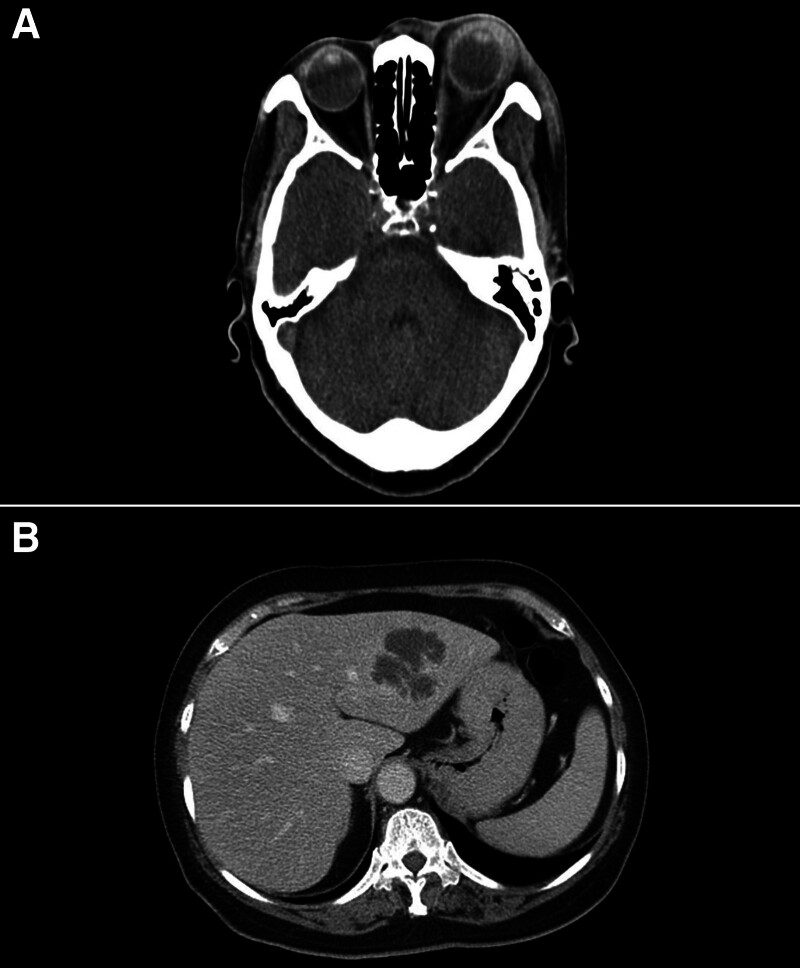
Computed tomography of the orbit (A) and liver abscess (B).

She had leukocytosis (white blood cell count 29,200/µL) with neutrophil predominance (91%) and mildly elevated liver enzymes (alanine transaminase at 66 U/L). Orbital computed tomography demonstrated subcutaneous swelling of the left eyelids, retrobulbar infiltration without orbital abscess, and scleral thickening with infiltration extending into the retrobulbar space (Fig. 2A). A B-scan of the eyeball revealed vitreous opacity with choroidal swelling.

To treat panophthalmitis, empirical intravenous antibiotics, including ceftriaxone and vancomycin, were initiated, along with intravitreal injection of ceftazidime. Debridement of necrotic tissue and cleansing of the pus and inflammatory debris were performed daily.

A liver ultrasound revealed a hypoechoic lesion measuring 5.1 cm in the left lobe of the liver, indicating a liver abscess, which was further confirmed by a computed tomography scan (Fig. 2B). Blood cultures taken on admission were negative, while Kp was isolated from the left eyelid abscess pus culture. Vancomycin was discontinued based on the culture results. Aspiration of the liver abscess also yielded Kp.

The patient’s condition continued to deteriorate. The eyelid abscess became more pronounced, and the orbit swelled with purulent discharge (Fig. 1B and C). The aforementioned systemic and intravitreal antibiotics were continued for 2 weeks.

After 2 weeks of treatment, the patient’s vision remained at no light perception. Throughout this entire period, she did not develop any systemic symptoms, such as fever or gastrointestinal disturbances. In the third week after admission, the patient finally agreed to evisceration. Following evisceration, the infection was controlled.

## 3. Discussion

Panophthalmitis is a rapidly progressing purulent process that involves the eye and the orbit, combining endophthalmitis and orbital cellulitis.^[1,2]^ Our case involves panophthalmitis caused by Kp originating from a liver abscess. Notably, most such cases exhibit systemic symptoms or clinical manifestations from other organ system. Fever and gastrointestinal symptoms are the most common. This case is particularly unusual and noteworthy because the patient exhibited almost no systemic symptoms, including fever and gastrointestinal disturbances. Being alert to such eye infections helps prevent the misdiagnosis of liver abscess.

In previous reports, most patients with panophthalmitis and extraocular abscess, such as the liver, lung, or kidney, had multiple underlying diseases, most commonly diabetes mellitus.^[3–7]^ In our case, the patient had no known systemic disease. Visual acuity at presentation was generally poor with only light perception, hand motion, or no light perception.^[3–7]^ Visual outcomes were generally poor across these cases.^[2–7]^

Endophthalmitis can be attributed to endogenous or exogenous sources. The latter is often secondary to eye trauma, corneal ulcers, or penetrating foreign bodies. Rarely does exogenous endophthalmitis cause fever. Our patient presented with severe endophthalmitis and orbital cellulitis and was referred from another regional hospital without a systemic survey. The only clue was the referral information that did not mention any periocular inflammation. If we had assumed the intraocular infection came from the periorbital contamination and did not seek the systemic sources of pathogen spreading, we might have missed the liver abscess and delayed the treatment. Despite the absence of fever or other systemic clinical manifestations, the presence of a large liver abscess, severe leukocytosis, and elevated liver enzymes all indicated a significant underlying infection. The potential risk of shock or death in this patient was substantial.

This case highlights several important points: first, severe infections can present with minimal or no systemic symptoms, underscoring the need for clinical vigilance in such scenarios. Second, the rapid progression from initial symptoms to panophthalmitis within just a few days illustrates the danger and rapid development of this condition. Third, although the patient’s vision did not recover, and evisceration was ultimately necessary—a poor prognosis consistent with previous literature—we successfully prevented the infection from spreading beyond the orbit. Additionally, the liver abscess was effectively managed with timely aspiration and systemic antibiotics, preventing further systemic complications. This indicates that timely diagnosis and appropriate treatment can achieve significant outcomes in controlling the spread of infection beyond the orbit.

To the best of our knowledge, this is the first reported case of Kp-related concurrent panophthalmitis and extraocular abscess that presented solely with ocular symptoms, without any systemic symptoms or clinical manifestations involving other organ systems. This case emphasizes the need for a high index of suspicion and thorough investigation in patients presenting with severe ocular symptoms, even in the absence of systemic signs. The importance of early and aggressive treatment cannot be overstated, as it may influence not only the survival of the eye but also the overall prognosis of the patient.

## 4. Conclusion

This case underscores the critical importance of maintaining a high index of suspicion for systemic infections in patients presenting with severe ocular symptoms, even in the absence of systemic manifestations such as fever or gastrointestinal complaints. The identification of an asymptomatic pyogenic liver abscess in a patient with Kp panophthalmitis highlights the insidious nature of certain infections and the potential for life-threatening complications if left undiagnosed. Thorough systemic evaluation, timely diagnosis, and prompt multidisciplinary management are essential not only for controlling the ocular infection but also for preventing systemic progression. Clinicians should be aware that ocular symptoms may serve as the initial or sole manifestation of serious underlying infections.

## Author contributions

**Conceptualization:** Shu-Han Chuang, Cheng-Hsien Chang.

**Investigation:** Shu-Han Chuang, Cheng-Hsien Chang.

**Methodology:** Shu-Han Chuang, Cheng-Hsien Chang.

**Validation:** Shu-Han Chuang, Cheng-Hsien Chang.

**Visualization:** Shu-Han Chuang.

**Writing – original draft:** Shu-Han Chuang.

**Funding acquisition:** Cheng-Hsien Chang.

**Project administration:** Cheng-Hsien Chang.

**Resources:** Cheng-Hsien Chang.

**Supervision:** Cheng-Hsien Chang.

**Writing – review & editing:** Cheng-Hsien Chang.
